# Durability and Additional Properties of Anodized Aluminum-Based Coatings with Different Wettability under Natural Conditions

**DOI:** 10.3390/ma16103729

**Published:** 2023-05-14

**Authors:** Klaudia Olkowicz, Kamil Kowalczyk, Zofia Buczko, Joanna Czwartos, Barbara Nasiłowska

**Affiliations:** 1 Aircraft Airworthiness Division, Air Force Institute of Technology, 01-494 Warsaw, Poland; kamil.kowalczyk@itwl.pl; 2 Łukasiewicz—Warsaw Institute of Technologies, 02-673 Warsaw, Poland; zofia.buczko@wit.lukasiewicz.gov.pl; 3 Institute of Optoelectronics, Military University of Technology, 00-908 Warsaw, Poland; joanna.czwartos@wat.edu.pl (J.C.); barbara.nasilowska@wat.edu.pl (B.N.)

**Keywords:** anodization, sealing, impregnation, contact angle, surface energy, corrosion, self-cleaning, anti-fouling, anti-icing

## Abstract

The study aimed to test the durability of coatings under natural conditions. The present study focused on the changes in wettability and additional properties of the coatings under natural conditions. The specimens were subjected to outdoor exposure and additionally immersed in the pond. Impregnating porous anodized aluminum is a popular production method for hydrophobic and superhydrophobic surfaces. However, prolonged exposure of such coatings to natural conditions causes leaching of the impregnate and, thus, the loss of hydrophobic properties. After the loss of hydrophobic properties, all kinds of impurities and fouling adhere better to the porous structure. Additionally, deterioration of anti-icing and anti-corrosion properties was observed. Finally, the self-cleaning, anti-fouling, anti-icing and anti-corrosion properties were comparable or even worse to those of the hydrophilic coating. In the case of superhydrophobic specimens, during outdoor exposure there was no loss of superhydrophobicity, self-cleaning and anti-corrosion properties. Still, despite this, the icing delay time dropped. During outdoor exposure, the structure, which initially had anti-icing properties, may degrade. Nevertheless, the hierarchical structure responsible for the superhydrophobic effect can still be preserved. The superhydrophobic coating initially had the best anti-fouling properties. However, the coating was also gradually losing its superhydrophobic properties during water immersion.

## 1. Introduction

Each surface is characterized by a specified wettability. Hydrophilic surfaces have a contact angle below 90°; hydrophobic surfaces have a contact angle of 90–150°. Superhydrophobic surfaces are characterized by contact angles higher than 150° and a sliding angle lower than 5°. Superhydrophobic surfaces are water-repellent, and this phenomenon is commonly used in waterproof fabrics [1]. In addition, water droplets sliding freely from the superhydrophobic surface collect accumulated dirt; therefore, the surface has excellent self-cleaning properties [2]. This is important for fabrics [1] and all surfaces used in architecture [2,3].

In the case of metal surfaces with superhydrophobic properties, they may be characterized by increased corrosion resistance [3,4,5]. This is due to the limited contact area between the superhydrophobic surface and the electrolyte. The small contact area between the solid and the water impedes heat transfer and thus delays ice formation; therefore, some superhydrophobic surfaces may also have good anti-icing properties [6,7]. In addition, superhydrophobic surfaces are used as antifouling and antibacterial surfaces [7,8,9], but not all superhydrophobic surfaces have these additional properties.

The above applications of superhydrophobic surfaces are basic; however, the available literature lists a much wider range of potential applications of surfaces characterized by contact angles higher than 150° [7,8,10].

The literature addresses many ways of obtaining superhydrophobic surfaces, which differ depending on the substrate material. In the review study by K. Ellinas et al. [7], the methods for obtaining superhydrophobic metal surfaces were divided into two general methods: obtaining a suitable roughness on the metal substrate or applying a superhydrophobic layer. A broad spectrum of different technologies was included in this general division. While obtaining superhydrophobic surfaces, researchers often try to replicate the lotus-leaf structure, which is a naturally occurring hierarchical structure with superhydrophobic properties [11,12,13,14]. In general, the production of a hierarchical structure characterized by multiscale roughness allows for superhydrophobic properties to be obtained [11,15]. The effect of superhydrophobicity may be achieved with an appropriate structure or by combining an appropriate structure and materials with low-surface energy [7].

Usually, the durability of the coatings and hydrophobic or superhydrophobic effect is determined during various abrasion tests and immersion in solutions of different pH [16,17,18,19,20,21,22,23,24,25,26,27,28]. The main aim of this study was to test the durability of aluminum-based coatings in natural conditions. In addition to the durability of the coatings, their self-cleaning, anti-fouling, anti-icing, and anti-corrosion properties were examined. Coatings based on the anodization process with different wettability characteristics were selected for the study.

One of the popular methods for aluminum surface protection is the anodization process. The anodization process can be carried out in sulfuric, phosphoric, oxalic, chromic and tartaric acids, among others [9,16,17,18,27,28,29,30,31,32,33,34,35]. However, in industrial practice, sulfuric acid is most often used. The unsealed aluminum oxide coating is porous and increases the adhesion of contaminations. For this reason, standard coatings obtained during anodizing for decorative and anti-corrosion purposes are sealed to close the pores. As part of this study, hydrophilic coatings were obtained during the basic anodization and sealing process [36,37].

Due to their porosity, it is also possible to impregnate the coatings with appropriate substances instead of standard sealing. The effectiveness of impregnation depends on the porosity and adhesion. Originally, in industrial practice, this procedure was aimed only at improving the tribological properties of anodized elements. Currently, this idea is increasingly used in the production of hydrophobic and superhydrophobic coatings [9,16,17,18,19,24,29,30,31,32,33,34,35,38,39,40,41]. Researchers often try to obtain a porous structure by modifying the anodization process and using the additional etching of the coating before impregnation [24,29,30,35]. The examples of hydrophobic and superhydrophobic coatings on aluminum achieved using the anodization process are given in Table 1. Table 1 only outlines the proposed processes without the details available in the cited literature. The resulting coatings have high porosity and are suitable for subsequent impregnation [24,29,39,42]. After impregnation with silicone, fatty acid, or polytetrafluoroethylene (PTFE), the coatings have hydrophobic or superhydrophobic properties [16,17,18,19,29,30,31,38,40,42]. As part of this study, hydrophobic coatings were obtained by modifying the anodization process in sulfuric acid to make the coating more porous. The coating was then impregnated with a suitable silicone.

In the case of superhydrophobic coatings, the present study focused on applying a combination of epoxy resin, SiO_2_ nanopowder, and silicone on a porous aluminum oxide coating. In the available literature, similar combinations of compounds were used to apply the coating directly to a non-porous substrate [25,43,44,45,46,47]. However, porosity increases the adhesion of applied materials. For this reason, in industrial practice, unsealed conversion coatings are used before applying paint or powder coatings to guarantee adhesion. Anodization prior to applying the combination of epoxy resin, SiO_2_ and silicone was used to increase the adhesion of the target superhydrophobic layer.

According to the literature review, the processes for obtaining individual coatings were selected to represent the most common ways of producing hydrophilic, hydrophobic and superhydrophobic coatings based on aluminum. The proposed production processes can be freely modified. In the presented study, research was undertaken to supplement the current state of knowledge on changes in the contact angle and on additional properties of this type of coating in natural conditions.

## 2. Experimental Methods

**Figure 1:**
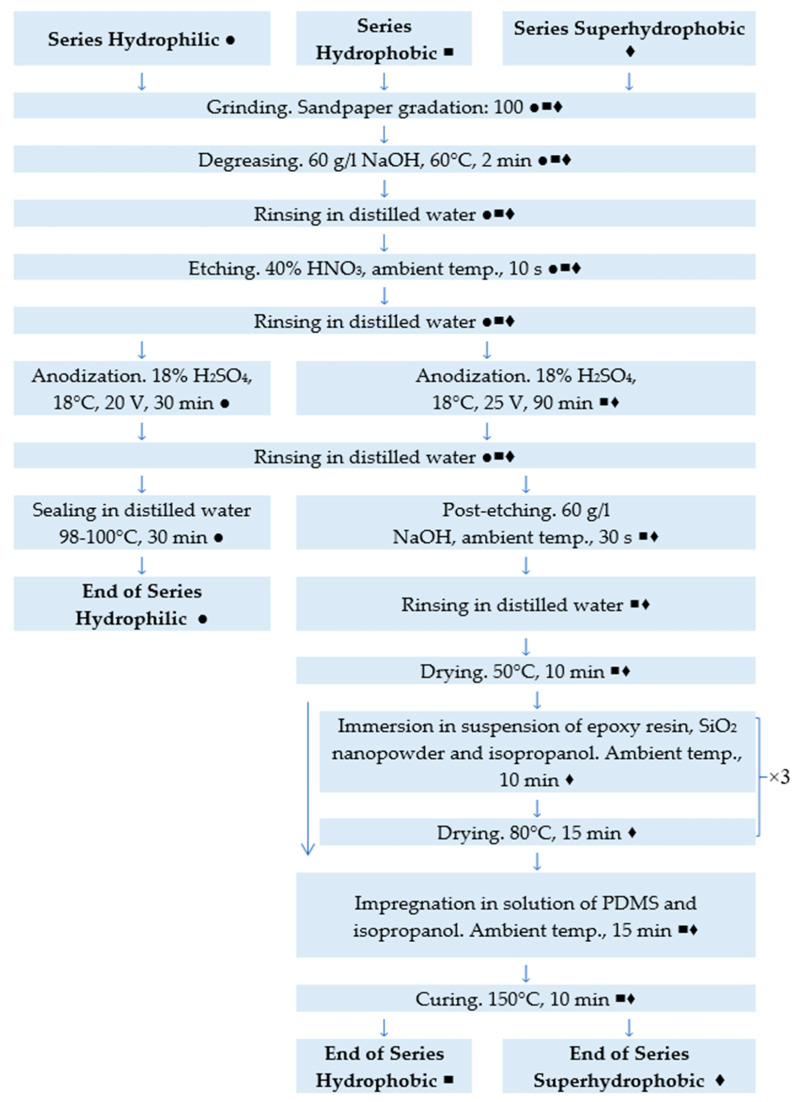
Specimen preparation scheme. ●—steps for Series Hydrophilic, ■—steps for Series Hydrophobic, ♦—steps for Series Superhydrophobic.

Specimens with dimensions of 50 mm × 30 mm × 3 mm were prepared on 1100 aluminum alloy (Kafra, Raszyn, Poland). Three different series of specimens were produced (Series Hydrophilic, Series Hydrophobic, and Series Superhydrophobic). The specimen preparation scheme is given in Figure 1. For all three series, the initial surface preparation and the anodization process were performed at the galvanizing plant of the Air Force Institute of Technology. Reagents for the preparation of solutions for degreasing, etching and anodization were supplied by Galvano-Partners (Lodz, Poland). The coatings on the specimens from Series Hydrophilic were obtained during the basic anodization process and then sealed in distilled water at 98–100 °C for 30 min.

In the case of Series Hydrophobic and Superhydrophobic, the anodization process was carried out at increased voltage and extended time. Then, the resulting coating was additionally etched in NaOH solution (Galvano-Partners, Lodz, Poland). The use of the current conditions during the anodization process and additional etching results in the development of the porous structure [24,29,40]. As a consequence, a coating with high wettability was created [9], which was beneficial for subsequent impregnation of the coating. Next, the specimens from Series Hydrophobic were impregnated with QLE 1102 two-component silicone elastomer system (CHT Germany GmbH, Tübingen, Germany). As the study of L. Cavas et al. indicates [48], QLE 1102 is a trade name for polydimethylsiloxane (PDMS). However, according to the safety data sheet, QLE 1102 has a lower viscosity and longer pot life than the most popular PDMS–Sylgrad 184. PDMS is often used to produce superhydrophobic coatings [8,9,23,33,43,47]. The silicone elastomer system was prepared by mixing component A with component B at a volume ratio of 1:1. The prepared PDMS was then mixed with isopropanol (Kontakt IPA Plus, TermaPasty, Sokoly, Poland) in a mass ratio of 1:37. The solution was stirred for 10 min at 500 rpm using a magnetic stirrer (uniSTIRRER 3, LLG GmbH, Meckenheim, Germany). The dried coating was then impregnated with the prepared solution for 15 min in an ultrasonic cleaner (Emmasonic P, Elma, Singen, Germany). The impregnated specimens were cured at 150 °C for 10 min.

In the case of Series Superhydrophobic, the specimens, after the anodization process and additional etching, were first covered with a suspension of SiO_2_ nanopowder and epoxy resin. SiO_2_ powder with a grain size of 5–20 nm was used for the experiment (Sigma-Aldrich, St. Louis, MO, USA). The epoxy resin (Epoxy resin 832C, MG Chemicals, Burlington, ON, Canada) was mixed with the curing agent at a volume ratio of 1:2. The epoxy resin prepared in this way was mixed with isopropanol, and then SiO_2_ was added. Epoxy resin, SiO_2_ nanopowder, and isopropanol were mixed in a mass ratio of 3:1:20. The resulting solution was stirred for 15 min using a magnetic stirrer at 500 rpm to form a suspension. Next, the specimens from Series Superhydrophobic were immersed in the prepared suspension in an ultrasonic cleaner for 10 min. Specimens were dried at 80 °C for 15 min immediately after removal from suspension. The application of epoxy resin and nanopowder was repeated three times. After the last application of epoxy resin and nanopowder, the specimens were impregnated with PDMS solution and cured at 150 °C as for the Series Hydrophobic specimens.

The surface of the specimens was tested using a scanning electron microscope (SEM). At first, the samples were plated with a 5 nm gold layer by sputtering (EMACE 600, Leica Microsystems, Inc., Wetzlar, Germany). Coating the specimens with a gold layer was intended to improve the conductivity of the samples, thereby improving the quality of the images taken by the SEM (Quanta 250 FEG SEM, FEI, Hillsboro, OR, USA).

The surface topography, profile, and roughness of specimens were examined using an atomic force microscope (NT-MDT Spectrum Instruments, Moscow, Russia). The measurements were performed at ambient conditions in semi-contact mode using a silicon AFM probe (HQ:NSC15/Al BS, MikroMasch^®^ SPM Probes&Test Structures, Watsonville, CA, USA) featuring a pyramidal tip with a curvature radius of ~8 nm. The cantilever of the probe was characterized by a constant force range from 20 to 80 N/m and a resonant frequency range of 265–410 kHz. The scan size of the collected topographies was 30 µm x 30 µm with a resolution of 256 points per line. The profile analysis and average surface roughness (S_a_) calculations were performed using Gwyddion 2.53 software.

The main aim of this study was to test the long-term durability of the specimens. Therefore, the specimens were placed on the atmospheric corrosion test rack to test the durability and purity of coatings and immersed in the pond to test the durability and anti-fouling properties. The atmospheric corrosion test rack and the pond are located at the Air Force Institute of Technology in Poland. Specimens were periodically examined for changes in contact angle and surface energy. No changes in the contact angle of the coatings proved the durability of the hydrophobic and superhydrophobic effects. Changes in surface energy were related to the durability of the self-cleaning properties of the specimens tested on an atmospheric corrosion test rack. In the case of specimens immersed in the pond, changes in surface energy were related to the fouling intensity of the specimens presented in the photographs. Three specimens from each series were tested on the atmospheric corrosion test rack and in the pond. Both tests were carried out from 1 June 2022 to 30 November 2022.

An optical microscope measured the contact angle (6000 VHX, Keyence Corporation, Osaka, Japan). The contact angle was measured by placing a drop of water on the surface of the tested coating and taking a picture. Then, the microscope software measured the contact angle based on the previously taken photo. Ten contact angle measurements were made for each specimen. The drop volume during measurements was 3 µL.

The surface energy of the coatings was measured using special measuring inks (SmartDrop-F, AcXys Technologies, Saint-Martin-le-Vinoux, France). The test uses the surface tension of a liquid to determine the surface energy of a material. The ink is applied on the tested surface. If the ink continuously spreads over the surface, the surface energy value is greater than the surface tension of the used ink. If the ink shrinks over the surface, the surface energy value is lower than the surface tension of the ink. The value of the surface energy of the tested material corresponds to the value of the ink which neither spreads nor shrinks when applied over the surfaces. The kit consists of inks with a measurement range of 28–72 mN/m. As the manufacturer declares, the inks are compliant with ISO 8296 standard.

The icing delay test used a Peltier plate (AST-TE C40-33-006 Advanced Thermal solutions, Norwood, MA, USA). The specimen with a waterdrop on the surface was placed on the Peltier plate and cooled from ambient temperature to −10 °C. The drop volume was 20 µL. The icing delay time was measured from the start of the cooling until the water was completely frozen. The complete freezing of water is manifested by the formation of characteristic peaks on top of a drop. Ten measurements were taken for each specimen.

An electrochemical corrosion test was performed using a potentiostat (SP-200, BioLogic, Orlando, FL, USA) in a three-electrode system. The specimen was a working electrode, the platinum mesh was a counter electrode, and the saturated calomel electrode was the reference electrode. The test was carried out in 3.5 wt.% NaCl at room temperature. The open-circuit potential was stabilized for 30 min. The corrosion current density (I_corr_) and potential (E_corr_) calculations were performed using EC-Lab software. Additionally, the percentage of corrosion inhibition efficiency (Ƞ_p_) was calculated based on the corrosion current densities.

## 3. Results and Discussion

**Figure 2:**
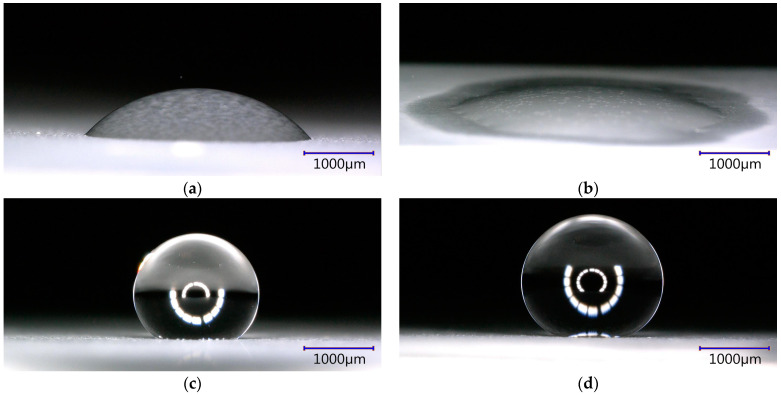
Wettability of specimen obtained during: (**a**) basic anodization process—Series Hydrophilic; (**b**) anodization at an increased voltage and extended time; (**c**) anodization at an increased voltage and extended time, and after impregnation by PDMS—Series Hydrophobic; and (**d**) anodization at an increased voltage and extended time, and after coating with epoxy resin, SiO_2_ nanopowder and PDMS—Series Superhydrophobic.

Three different series of specimens were produced—hydrophilic, hydrophobic, and superhydrophobic (Figure 2). The average contact angle of the specimens was 26° ± 8°, 139° ± 6° and 155° ± 2° respectively. The coating obtained during anodization at the increased voltage and extended time before impregnation was superhydrophilic (Figure 2b). The thickness of the coatings from Series Hydrophilic, Hydrophobic and Superhydrophobic was 13.6 ± 0.5, 32.1 ± 1.5 and 37.8 ± 1.5 µm, respectively.

### 3.1. SEM and AFM Analysis

**Figure 3:**
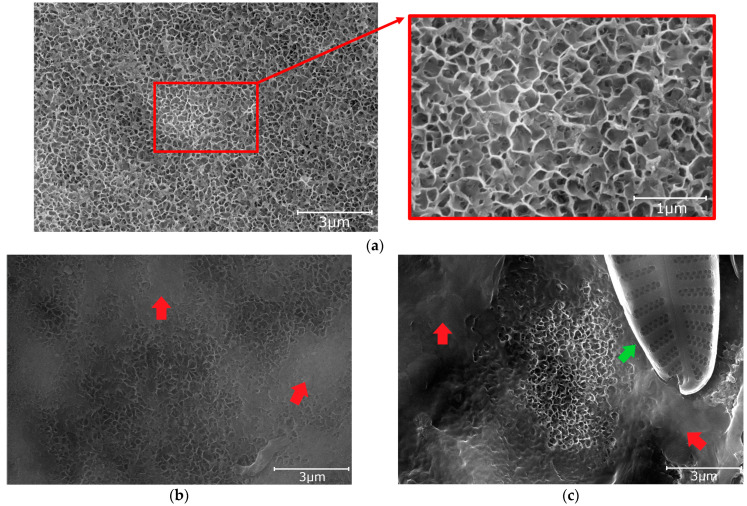
The surface of the specimens from Series Hydrophilic (SEM): (**a**) initial; (**b**) after test on the atmospheric corrosion test rack; and (**c**) after test in the pond.

**Figure 4:**
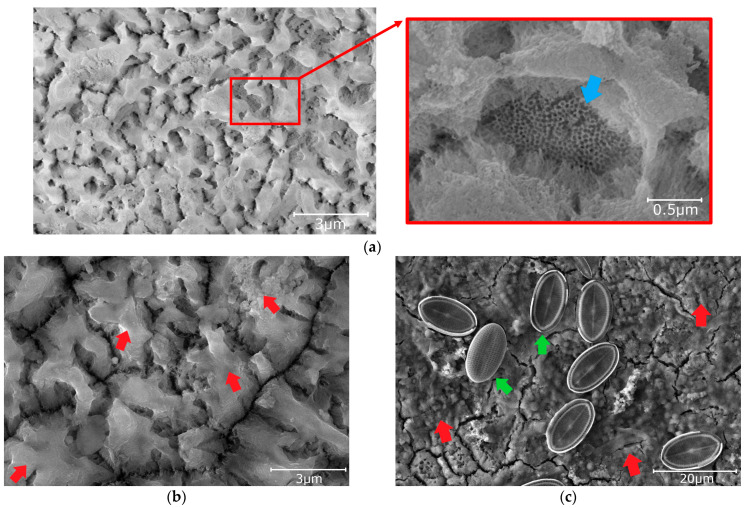
The surface of the specimens from Series Hydrophobic (SEM): (**a**) initial; (**b**) after test on the atmospheric corrosion test rack; and (**c**) after test in the pond.

**Figure 5:**
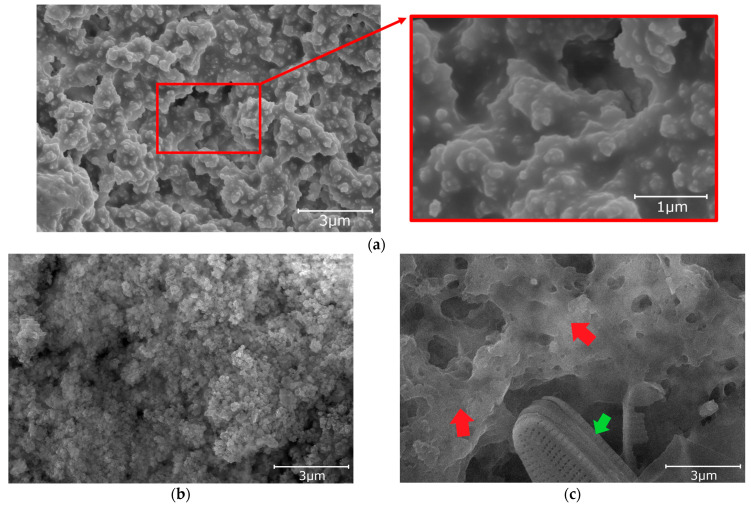
The surface of the specimens from Series Superhydrophobic (SEM): (**a**) initial; (**b**) after test on the atmospheric corrosion test rack; and (**c**) after test in the pond.

Pictures of specimens taken by scanning electron microscopy are shown in Figure 3, Figure 4 and Figure 5. The Figures show the initial structure of the specimens and the structure after tests under natural conditions. The structure of the aluminum oxide coating after sealing the specimen from Series Hydrophilic is given in Figure 3a. During the anodization process, a porous aluminum oxide coating is formed. The sealing process is intended to close the pores of the aluminum oxide coating and thus increase the corrosion resistance of the coating. Sealing in hot water causes the hydration of aluminum oxide and the formation of aluminum hydroxide oxide. During the initial phase of sealing in hot water, the pores of the oxide only increase in volume and decrease in diameter. However, as further sealing is carried out, aluminum oxide hydroxide precipitates on the surface of the coating in the form of characteristic flakes [36,37,49,50]. In the case of the coating from Series Hydrophilic, the pores of the coating were invisible after sealing because they were under the layer of aluminum hydroxide oxide. Typically, the pores of this type of coating before sealing have diameters of 12–20 nm [9,51,52].

The oxide coating of Series Hydrophobic and Superhydrophobic obtained during anodization at an increased voltage, extended time, and additional etching were characterized by a spongy structure (Figure 4). The pores, which were not fully etched, were preserved only in the cavities of the coating (blue arrow—Figure 4a). These pores were characterized by a diameter of 30–40 nm. The cavities in the coating are in the range of 500–1500 nm. This structure is typical of oxide coatings obtained during anodization in sulfuric acid at elevated voltage and extended time (Figure 4a). A similar coating structure (Figure 4a) was obtained in the available literature [17,38,40,43]. These coatings had high wettability before impregnation (Figure 2b). Any modifications to the anodization process and additional etching were aimed at obtaining a superhydrophilic structure suitable for further impregnation. Suitable impregnation of the initially superhydrophilic structure resulted in a hydrophobic or superhydrophobic coating at the final stage (Figure 2c) [24,29,30,31,35,39,40,42].

Coatings from Series Superhydrophobic were first immersed in a suspension of epoxy resin, SiO_2_ nanopowder, and isopropanol. After curing, additional impregnation with PDMS was applied. Applying the epoxy resin and SiO_2_ nanopowder resulted in complete coverage of the oxide coating (Figure 5a). The epoxy resin and SiO_2_ nanopowder settled on the coating in clusters and formed a hierarchical structure. Similar processes have been used in the literature and are indicated as durable solutions [25,43,44,45,46]. The obtained structure from Series Superhydrophobic (Figure 5a) observed with SEM was similar to those in available literature [25,43,44,45,46].

The structure of the specimens after tests under natural conditions is given in Figure 3b,c, Figure 4b,c and Figure 5b,c. When comparing the structures of the coatings after the test on the atmospheric corrosion test rack with the initial structures, it can be seen that the structure of specimens from Series Hydrophilic and Hydrophobic is partially covered with dirt (red arrows—Figure 3b and Figure 4b). In the specimen from Series Superhydrophobic, no dirt was observed on the surface, but the structure after the test became finer (Figure 5b). After the pond test, the structures of all specimens were covered with fouling (red arrows—Figure 3c, Figure 4c and Figure 5c). Additionally, microorganisms can be observed on the surface of the specimens (green arrows—Figure 3c, Figure 4c and Figure 5c). The microorganisms visible in the Figures are diatoms [53].

The average surface roughness of the specimens was examined using an AFM. Specimens from Series Superhydrophobic were characterized by the highest roughness of all the specimens, due to the manufacturing process. The average surface roughness examined using an AFM of the specimens from Series Hydrophilic was 451.5 nm. In the available literature, the following surface roughness values of 918.4 nm [54]; 213 nm [55]; 213 nm [55]; 342 nm [56] were obtained for anodized aluminum. In the case of polishing the substrate before anodization, the average obtained roughness values were lower [55,56]. The average surface roughness of the specimens from Series Hydrophobic was 284.9 nm. In the case of similar coatings from the available literature, the roughness value of 196 nm [17], 134 nm [38], 168.33 nm [40] and 219.4 nm [19] were obtained. The average surface roughness of the specimens from Series Superhydrophobic was 650.9 nm. Coatings similar to the specimens from Series Superhydrophobic were characterized by the following values of average surface roughness: 153.42 nm [49], 176.85 nm [49], 188.18 [49]; 359 nm [23] and 229.5 nm [57].

**Figure 6:**
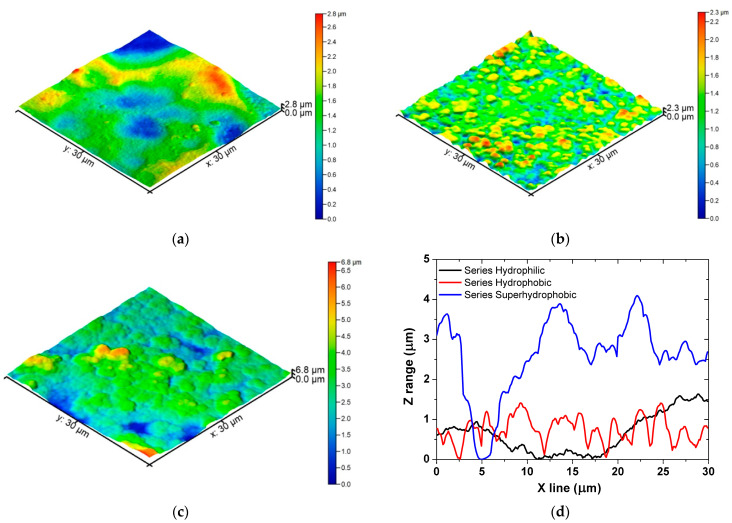
AFM topography of the specimens: (**a**) Series Hydrophilic; (**b**) Series Hydrophobic; (**c**) Series Superhydrophobic; and (**d**) profiles of the specimens obtained from AFM topographies along the selected line.

Examination of the surface of the specimens using atomic force microscopy (AFM) gives not only the information about the roughness values of the material, but also the information about the profiles (Figure 6), i.e., information about the height of the structures that occurred on the substrate of the aluminum. Thus, in the case of Series Hydrophilic, the structures formed did not exceed 1.7 µm in height (Figure 6a,d). The structures from Series Hydrophobic did not exceed 1.5 µm in height (Figure 6b,d). However, in this case, the coating formed was characterized by a quasi-regular repeatability of the structures. As for the specimens from Series Superhydrophobic, the resulting structures were ~2.5 times higher compared to the previous two Series, and their heights did not exceed 4.2 µm (Figure 6c,d). The depths of cavities measured for this case ranged from ~2 um to as much as ~4 µm.

### 3.2. Durability, Purity and Anti-Fouling Properties of the Coatings

**Figure 7:**
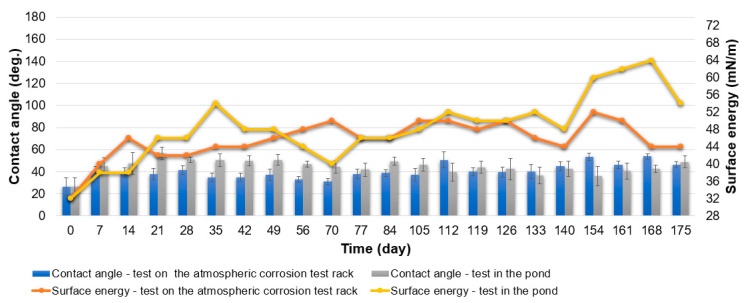
Specimens from Series Hydrophilic. Contact angle and surface energy after test on the atmospheric corrosion test rack and test in the pond.

**Figure 8:**
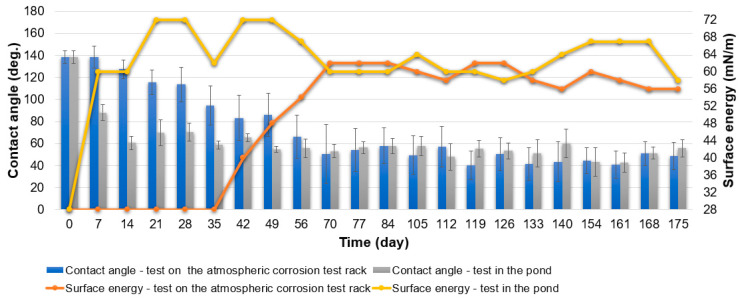
Specimens from Series Hydrophobic. Contact angle and surface energy after test on the atmospheric corrosion test rack and test in the pond.

**Figure 9:**
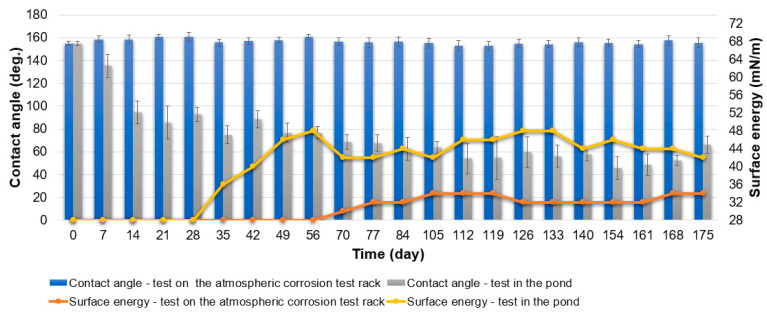
Specimens from Series Superhydrophobic. Contact angle and surface energy after test on the atmospheric corrosion test rack and test in the pond.

The specimens were placed on the atmospheric corrosion test rack and in the pond. The study focused on the durability, purity and anti-fouling properties of the coatings by measuring their contact angle and surface energy. The climate (temperature, humidity and precipitation) during the tests is shown in Figure S1. The tests were carried out from June to November in a temperate climate, in which the specimens were exposed to various weather conditions. The test results are given in Figure 7, Figure 8 and Figure 9.

In the case of specimens from Series Hydrophilic during the test on the atmospheric corrosion test rack, the initial value of the contact angle was 26°. Thereafter, the contact angle increased and remained in the range of 35–50° for most of the test (Figure 7). The surface energy of the specimens from Series Hydrophilic initially increased and then ranged from 42 to 52 mN/m (Figure 7).

The contact angle of specimens from Series Hydrophobic during the test on the atmospheric corrosion test rack decreased gradually (Figure 8). The hydrophobic properties of the specimens lasted up to 35 days of the test. After the 35th day of the test duration, the contact angle decreased below 90°. At the end of the test, the contact angle was about 40° and was similar to the contact angle of the Series Hydrophilic specimens. The surface energy remained at 28 mN/m as long as the contact angle was above 90° (Figure 8). This means the surface had excellent self-cleaning properties as long as the specimens had hydrophobic properties. As the contact angle decreased further, the surface energy of the specimens increased. Between the 70th and 175th days of the test, the surface energy remained in a range of 56–62 mN/m (Figure 8). The test demonstrated that the specimens from Series Hydrophobic have low durability in natural conditions. After losing their hydrophobic properties, they are characterized by lower purity than aluminum anodized by the basic method (specimens from Series Hydrophilic). It is caused by the intensive leaching of silicone from the pores of the coating during the test under natural conditions. The coating obtained during anodization at the increased voltage and extended time was characterized by superhydrophilicity and increased porosity; only impregnation with PDMS (or other low surface energy substance) resulted in obtaining hydrophobic or superhydrophobic properties [9,16,19,24,34,35,38,39,40]. Impregnating porous substrates is a popular way to obtain hydrophobic and superhydrophobic coatings, but it has proven to be unstable with longer testing periods.

The specimens from Series Superhydrophobic retained their superhydrophobic properties throughout the test on the atmospheric corrosion test rack, demonstrating high durability (Figure 9). The contact angle did not decrease below 150° during the test. The surface energy of the specimen from Series Superhydrophobic increased slightly after 56 days of the test and remained in a range of 32–34 mN/m, which proves the excellent self-cleaning properties of the specimens (Figure 9). Surface energy increased slightly after 70 days of testing; however, the specimens from Series Superhydrophobic were still characterized by the best self-cleaning properties compared to other coatings. The purity of the specimens from Series Superhydrophobic was also confirmed by scanning electron microscopy (Figure 5b). SEM images show dirt adhering to the surface of the specimens from Series Hydrophilic and Hydrophobic (red arrows—Figure 3b and Figure 4b).

The second set of specimens was placed in the pond. The specimens were periodically removed from the water and air-dried. During the tests, changes in the contact angle and surface energy were measured, and photographs were taken to document the fouling process.

**Figure 10:**
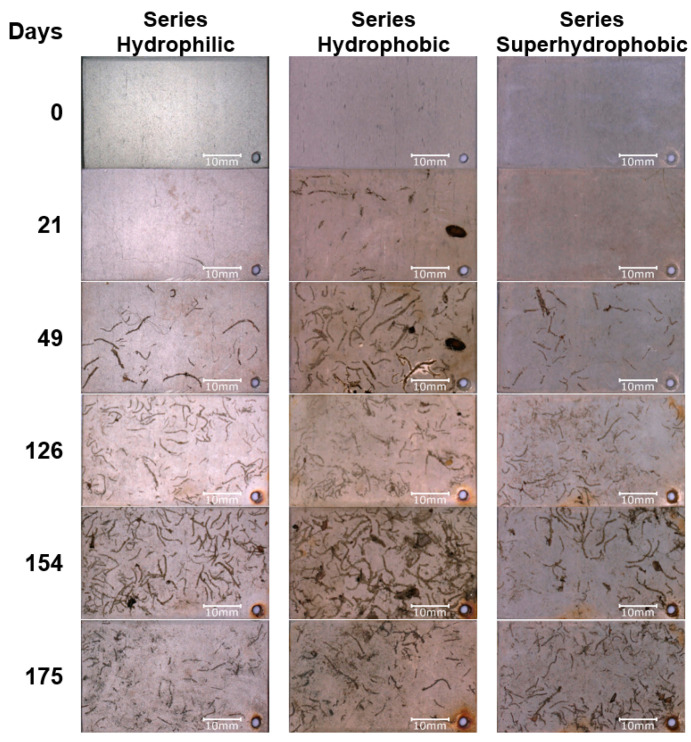
The examples of specimens during testing of anti-fouling properties.

In the case of specimens from Series Hydrophilic during the test in the pond, the contact angle remained in a range of 35–50°, as was the case with the Series Hydrophilic specimens exposed to natural conditions on the atmospheric corrosion test rack (Figure 7). By the 28th day of the test, the surface energy of the specimens gradually increased, and only a little dirt accumulated on the surface of the specimens (Figure 10). The surface energy between the 35th and 140th days of the test in the pond remained in a range of 40–54 mN/m, and the specimens began to overgrow more intensively during this time. Between the 140th and 168th days, the fouling intensity and the surface energy value of the specimens increased even more (Figure 7 and Figure 10).

Series Hydrophobic specimens lost their hydrophobic properties very quickly during anti-fouling tests in the pond (Figure 8). After seven days, the contact angle decreased below 90°; at the same time, the surface energy increased to 60 mN/m, and dirt adhered to the surface of the specimens from Series Hydrophobic (Figure 10). During the test, the surface energy changed and remained in a range of 58–72 mN/m (Figure 8). The surface of the specimen was overgrown intensively. The specimens from Series Hydrophobic had worse anti-fouling properties than those from Series Superhydrophobic, which was also confirmed by photographic documentation (Figure 10). Initially, the specimens from Series Hydrophilic had better anti-fouling properties than the specimens from Series Hydrophobic. However, at the end of the test, the fouling intensity of the specimens from Series Hydrophilic and Hydrophobic was similar.

In the case of the specimens from Series Superhydrophobic, the contact angle decreased gradually during the test in the pond (Figure 9). After 112 days of the test, the contact angle stabilized and remained in a range of 45–60°. Initially, the specimens from Series Superhydrophobic had the best anti-fouling properties. The surface energy remained at 28 mN/m as long as the contact angle was above 90°, indicating the non-fouling of the specimens. After 28 days of the test, the contact angle decreased below 90°. Between the 28th and 56th day of the test, the surface energy of the Series Superhydrophobic specimens gradually increased, and with it, the specimens began to overgrow. Then, the surface energy stabilized and ranged from 42 to 48 mN/m between the 56th and 175th days of the test. The fouling intensity of the Series Hydrophilic and Superhydrophobic specimens was also similar between the 56th and 140th days of the test, as confirmed by photographic documentation (Figure 10). However, after 140 days of the test, the specimens from Series Superhydrophobic had better anti-fouling properties.

In the last week of the test, fouling intensity and surface energy decreased for all specimens. This was caused by the decrease in temperature below 0 °C (Figure S1) and, thus, less biological activity in the pond.

In addition to verifying the self-cleaning and anti-fouling properties, the tests perfectly exposed the durability of the hydrophobic and superhydrophobic effects under natural conditions. The conducted tests and the literature data (Figure 8, Table S1) confirm that coatings obtained during the anodization and impregnation process often lose their hydrophobic and superhydrophobic properties very quickly under natural conditions. In the case of coatings based on SiO_2_, epoxy resin and PDMS, it was confirmed that these types of coatings have high durability. However, prolonged water immersion can cause the loss of superhydrophobic properties, which is also confirmed by literature data (Table S1). The available literature confirms that coatings based on PDMS or other silicones have potential anti-fouling properties—good results are achieved by directly applying silicone to the substrate [8,48,58,59]. However, in the case of direct impregnation of porous structures, there is a risk of rapid leaching of the impregnate and, thus, loss of self-cleaning and anti-fouling properties.

It should be noted that the problem of surface contact-angle changes under natural conditions is complex. As mentioned before, the impregnate is often degraded or leached during tests in natural conditions. Despite the decrease in the initial contact angle, the self-cleaning and anti-fouling properties of the coatings can be preserved as long as the contact angle is above 90°. When the contact angle decreases below 90°, dirt gradually begins to adhere to the surface. Impurities and fouling are slowly beginning to cover the original nanostructure of the surface, which was confirmed by SEM images (red arrows—Figure 3b,c, Figure 4b,c and Figure 5c). In addition, after degradation or leaching the impregnate, all kinds of contaminants adhered better to the coatings due to the exposure of the porous structure of the coating, which had hydrophilic or superhydrophilic properties before impregnation. Covering structures with impurities and fouling (and their potential hydrophilic properties) may additionally intensify surface wettability.

### 3.3. Anti-Icing Delay

**Figure 11:**
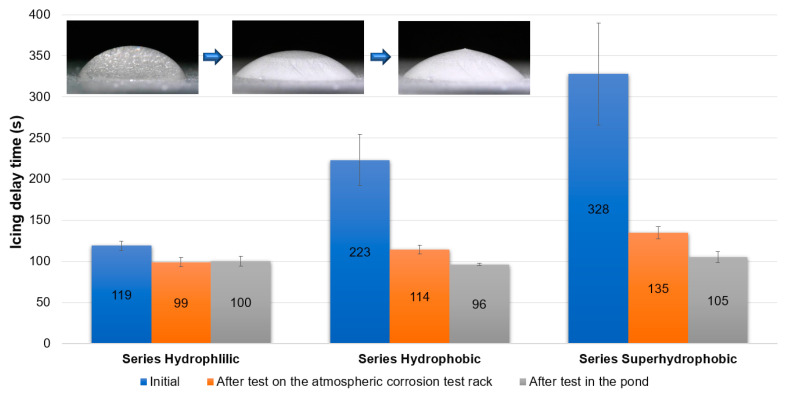
Icing delay time for the specimens.

Icing delay time (Figure 11) was measured from the start of the cooling until the water was completely frozen. The specimens were cooled from ambient temperature to −10 °C for 108 s ± 6 s. A temperature of 0 °C was reached in 49 s ± 2 s.

After the preparation process, the specimens from Series Superhydrophobic were characterized by the best icing delay time. The data of the specimens from Series Hydrophobic and Superhydrophobic were close to values from the available literature, although there are some samples with better anti-icing properties (Table S2). However, after testing under natural conditions, the anti-icing properties of all specimens deteriorated significantly. The icing delay time was low and ranged from 96 to 119 s. For some specimens after tests under natural conditions, the water during the test froze before reaching −10 °C, usually at −8 °C. In the case of the specimens from Series Hydrophobic after tests on the atmospheric corrosion test rack and in the pond, and from Series Superhydrophobic after test in the pond, the impregnate was leached. A decrease in the contact angle below 90° caused the loss of the self-cleaning and anti-fouling properties. After degradation or leaching the impregnate, all kinds of contaminants adhere better to the coatings (red arrows—Figure 4b,c and Figure 5c). The presence of impurities on the structure and the exposure of a structure with increased porosity (in the case of Series Hydrophobic) cause water to spread over the surface. This also has consequences in the form of deterioration of anti-icing properties (Figure 7, Figure 8, Figure 9 and Figure 11).

After the test on the atmospheric corrosion test rack, the specimens from Series Superhydrophobic were still characterized by the contact angle above 150°, but the icing delay time dropped despite this. The icing delay time of the specimen from Series Superhydrophobic after the test on the atmospheric corrosion test rack was admittedly the best among the other specimens after testing under natural conditions. However, this value was low in comparison with the initial icing delay time and literature data (Table S2, Figure 11). It should be noted that despite the durability of the superhydrophobic properties, the anti-icing properties of the coating may change during prolonged exposure to natural conditions. As indicated in the available studies, the structure, which initially had anti-icing properties, may degrade [60,61,62]. Nevertheless, the hierarchical structure responsible for the superhydrophobic effect can still be preserved. The described structure changes were visible in the SEM images. The structure of the specimen from Series Superhydrophobic became finer after the test under natural conditions (Figure 5a,b).

### 3.4. Corrosion Resistance

**Figure 12:**
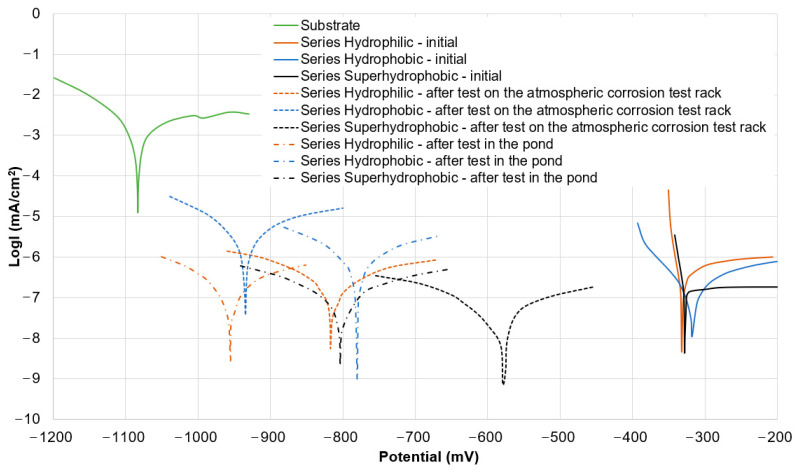
The potentiodynamic polarization curves of the specimens.

Table 2 and Figure 12 show the results of the electrochemical corrosion test. A surface with a lower corrosion current density (I_corr_) value and a higher corrosion potential (E_corr_) value is characterized by better corrosion resistance. Regardless of the coating wettability, all specimens were initially characterized by similar corrosion resistance. The corrosion inhibition efficiency (Ƞ_p_) for initial specimens of all three series was 100%. The corrosion current densities (I_corr_), potentials (E_corr_) and inhibition efficiencies (Ƞ_p_) values of initial specimens from Series Hydrophilic, Hydrophobic and Superhydrophobic are similar to some literature values (Table S3).

Analyzing the potentiodynamic polarization curves and the I_corr_ and E_corr_ values, in the case of the specimens after the test on the atmospheric corrosion test rack, the specimen from Series Superhydrophobic had the best corrosion resistance due to the preservation of the contact angle above 150°. In contrast, the specimens from Series Hydrophobic after tests under natural conditions very quickly lost their hydrophobic properties, thus it was also characterized by worse corrosion resistance. In general, the specimens from Series Superhydrophobic had the best corrosion resistance of all the specimens after testing under natural conditions (Table 2, Figure 12).

In general, the corrosion potential of the specimens after tests under natural conditions has shifted toward negative values compared to the initial specimens. However, the I_corr_ and E_corr_ values of the specimens after tests under natural conditions are still much better than in the case of the bare aluminum substrate (Table 2, Figure 12). Despite differences in the corrosion current densities (I_corr_) and corrosion potentials (E_corr_) values, all specimens were characterized by high corrosion inhibition efficiency (Ƞ_p_) in the range of 99.83–100%, even after tests under natural conditions (Table 2). Compared to the literature data, this was a very good result (Table S3). The decrease in the corrosion inhibition efficiency was visible for specimens from Series Hydrophobic after tests in natural conditions (compared to the other specimens). After leaching the impregnate and losing hydrophobic properties, all kinds of contaminants adhere better to the coatings due to the exposure of the porous structure of the coating causing the electrolyte to freely spread over the surface and have free access to the porous structure, which deteriorates the anti-corrosion properties.

Series Superhydrophobic specimens, after testing in the pond, were still characterized by high values of corrosion inhibition efficiency. The specimens from Series Superhydrophobic were obtained by applying a combination of epoxy resin, SiO_2_ nanopowder and silicone on a porous oxide coating. When the silicone was leached during the test in the pond, the superhydrophobic properties were lost, but the porous structure was still obscured by the mixture of epoxy resin and nanopowder. This made it difficult for the electrolyte to penetrate the porous coating. In this case, the loss of superhydrophobic properties did not affect the deterioration of corrosion resistance.

## 4. Conclusions

The present study focused on the durability and additional properties of the coatings with different wettability under natural conditions. The hydrophilic coating was obtained through standard anodization and sealing. The hydrophobic coating was obtained during the impregnation of porous anodized aluminum with silicone. The superhydrophobic surface was obtained by applying a combination of epoxy resin, SiO_2_ nanopowder, and silicone on a porous anodized aluminum. The specimens were subjected to outdoor exposure and additionally immersed in the pond. Hydrophobic coatings obtained by the impregnation of porous anodized aluminum are not durable in natural conditions, as opposed to superhydrophobic coatings. As a result of our comparative studies, the following findings are presented:

- The hydrophobic and superhydrophobic coatings had excellent self-cleaning and anti-fouling properties as long as the contact angle of the surface was above 90°;
- Long-term exposure to natural conditions causes leaching the impregnate from the pores of the hydrophobic coating. After leaching the impregnate and losing hydrophobic properties, all kinds of contaminants adhere better to the porous structure. The exposure of a structure with increased porosity and the presence of the impurities on the structure cause the electrolyte to freely spread over the surface and have free access to the porous structure, which also deteriorates the anti-icing and anti-corrosion properties;
- The additional properties of the hydrophobic coatings after leaching of the impregnate are comparable to, or even worse than, those of the hydrophilic coating;
- No loss of superhydrophobicity, self-cleaning, and anti-corrosion properties was observed during the outdoor exposure of the superhydrophobic coating. Only the icing delay time deteriorated. During outdoor exposure, the structure, which initially had anti-icing properties, may degrade. Nevertheless, the hierarchical structure responsible for the superhydrophobic effect can still be preserved;
- During immersion in the pond, the superhydrophobic specimens initially had the best anti-fouling properties. However, prolonged water immersion may cause a loss of hydrophobic properties.
- The superhydrophobic coatings, after the test in the pond, were still characterized by high values of corrosion inhibition efficiency. Even with the loss of superhydrophobic properties, the porous structure was still obscured by a combination of epoxy resin and SiO_2_ nanopowder, which made it difficult for the electrolyte to penetrate the porous coating.

## Figures and Tables

**Table 1:** Examples of hydrophobic and superhydrophobic coatings on aluminum obtained by anodization.

LP	Pre-Treatment	Anodization	Post-Treatment	Contact Angle	Ref.
1	Abrasive-blasting; Degreasing in NaOH	H_2_SO_4_; 5 A/dm^2^; 25 °C; 50 min	Impregnation by myristic acid in ethanol	162°	[16]
2	Degreasing in NaOH; Etching in HCl	H_2_SO_4_; 6.25 A/dm^2^; ambient temp.; 60 min	Impregnation by lauric acid in ethanol, Rinsing in ethanol; Drying	154°	[17]
3	Abrasive-blasting; Degreasing in NaOH	H_2_SO_4_; 4.5 A/dm^2^; 20 °C; 45 min	Additional etching in NaOH; Impregnation by PDMS; Rinsing in toluene; Curing	159°	[9]
4	Polishing	H_2_SO_4_; 15 V; ambient temp., 25 min	Impregnation by KH-832 silane in ethanol; Heating	170°	[38]
5	Annealing; Mechanical polishing; Electropolishing	H_2_C_2_O_4_; 30 V; 25 °C; 3 h	Additional etching in H_3_PO_4_; Impregnation by PFODA in ethanol; Drying	161.5°	[24]
6	Electropolishing	H_3_PO_4_; 120 V; 50 °C; 20 min	Impregnation by POTS in ethanol; Heating	167.1°	[18]
7	Degreasing in acetone; Electropolishing	First anodization: H_2_C_2_O_4_; 40 V; 20 °C; 10 h Coating removal: H_3_PO_4_; H_2_CrO_4_; 65 °C; 10 h Second anodization: H_2_C_2_O_4_; 40 V; 20 °C; 160 s	Additional etching in H_3_PO_4_; cleaning by O_2_; Degreasing in acetone and methanol; Drying; Spin-coating by PTFE solution; Baking	139°	[29]
8	Degreasing in acetone	H_3_PO_4_; 50 V; 18 °C; 90 min	PTFE sputtering	165°	[32]
9	Polishing	H_3_PO_4;_ 50 V; 18 °C; 90 min	Impregnation by PTFE suspension; Heating	154.7°	[39]
10	Mechanical polishing; Degreasing in NaOH; Etching in HNO_3_	H_2_SO_4_; 16 V; 25 °C; 60 min	Impregnation by molten myristic acid; Rinsing in toluene; Drying	155.6°	[40]
11	Mechanical polishing; Degreasing in NaOH; Etching in HNO_3_	H_2_SO_4_; 20 V; 25 °C; 60 min	Impregnation by molten myristic acid; Rinsing in toluene; Drying	155.2°	[19]
12	Degreasing in acetone; Electropolishing	First anodization: H_3_PO_4_; 175 V; 0 °C; 60 min Coating removal: H_3_PO_4_; H_2_CrO_4_; 60 °C Second anodization: H_2_C_2_O_4_; 175 V; 0 °C; 15–300 min	Additional etching in H_3_PO_4_; Impregnation by FDTS; Heating	146°	[30]
13	Mechanical polishing; Degreasing in NaOH; Etching in HNO_3_	H_2_SO_4_; 25 V; 0 °C; 60 min	Impregnation by stearic acid; Rinsing in ethanol; Drying	152°	[31]
14	Mechanical polishing; Degreasing in acetone and ethanol	H_2_C_2_O_4_; 0.5 A/cm^2^; ambient temp.; 12 min	Impregnation by PDMS and SiO_2_ suspension; Drying	158°	[33]
15	Annealing; Mechanical polishing; Degreasing in acetone	H_2_SO_4_; 32 A/dm^2^; 21–27 V; 25 °C; 7 min	Impregnation by molten myristic acid; Rinsing in ethanol; Drying	154°	[34]
16	Mechanical polishing; Degreasing in acetone and NaOH; Etching in HNO_3_	H_2_SO_4_; C₆H₈O₇; 2.25 A/dm^2^; 25 °C; 1 h	Additional etching in CrO_3_ solution; Impregnation by KH-832 silane in ethanol; Heating	167.7°	[35]

**Table 2:** The corrosion current density and corrosion potential of the specimens.

Specimen	I_corr_ (A/cm^2^)	E_corr_ (mV)	Ƞ_p_ (%) *
Substrate	3.5 × 10^−6^	−1087	–
Series Hydrophilic—initial	1.4 × 10^−10^	−334	100
Series Hydrophobic—initial	2.5 × 10^−11^	−317	100
Series Superhydrophobic—initial	2.5 × 10^−11^	−327	100
Series Hydrophilic—after test on the atmospheric corrosion test rack	4.9 × 10^−10^	−811	99.99
Series Hydrophobic—after test on the atmospheric corrosion test rack	6.0 × 10^−9^	−931	99.83
Series Superhydrophobic—after test on the atmospheric corrosion test rack	7.5 × 10^−11^	−579	100
Series Hydrophilic—after test in the pond	4.0 × 10^−10^	−952	99.99
Series Hydrophobic—after test in the pond	2.0 × 10^−9^	−784	99.94
Series Superhydrophobic—after test in the pond	1.8 × 10^−10^	−806	99.99

* ${Ƞ}_{p}=\frac{\left(I_{corr\;subsrate}-I_{corr}\right)}{I_{corr\;substrate}}\cdot 100\%$.

## Data Availability

The data presented in this study are available upon request from the corresponding author.

## Supplementary Materials

The following supporting information can be downloaded at: https://www.mdpi.com/article/10.3390/ma16103729/s1. Figure S1: The climate in Warsaw during the tests (1 June 2022–30 November 2022); Table S1: Durability of the hydrophobic and superhydrophobic coatings from the available literature; Table S2: Icing delay time of coatings from the available literature; Table S3: Potentiodynamic polarization parameters for hydrophilic, hydrophobic and superhydrophobic coatings from the available literature. References [63,64,65,66,67,68,69,70,71,72,73,74,75,76,77,78] are cited in the Supplementary Materials.
