# Supplementary material for: Durability and Additional Properties of Anodized Aluminum-Based Coatings with Different Wettability under Natural Conditions
Source: Materials (Basel). 2023 May 14;16(10):3729. doi: 10.3390/ma16103729 (PMC10221504; doi:10.3390/ma16103729)
Supplement: Supplementary file 1 [file materials-16-03729-s001.zip › materials-2373178-supplementary.pdf]

materials

Supplementary Materials

Durability and Additional Properties of Anodized Aluminum-Based Coatings with Different Wettability under Natural Conditions

Klaudia Olkowicz [1]*, Kamil Kowalczyk [1], Zofia Buczko [2], Joanna Czwartos [3] and Barbara Nasiłowska [3]

[1] Aircraft Airworthiness Division, Air Force Institute of Technology, 01-494 Warsaw, Poland; kamil.kowalczyk@itwl.pl
[2] Łukasiewicz—Warsaw Institute of Technologies, 02-673 Warsaw, Poland; zofia.buczko@wit.lukasiewicz.gov.pl
[3] Institute of Optoelectronics, Military University of Technology, 00-908 Warsaw, Poland; joanna.czwartos@wat.edu.pl (J.C.); barbara.nasilowska@wat.edu.pl (B.N.)
* Correspondence: klaudia.olkowicz@itwl.pl

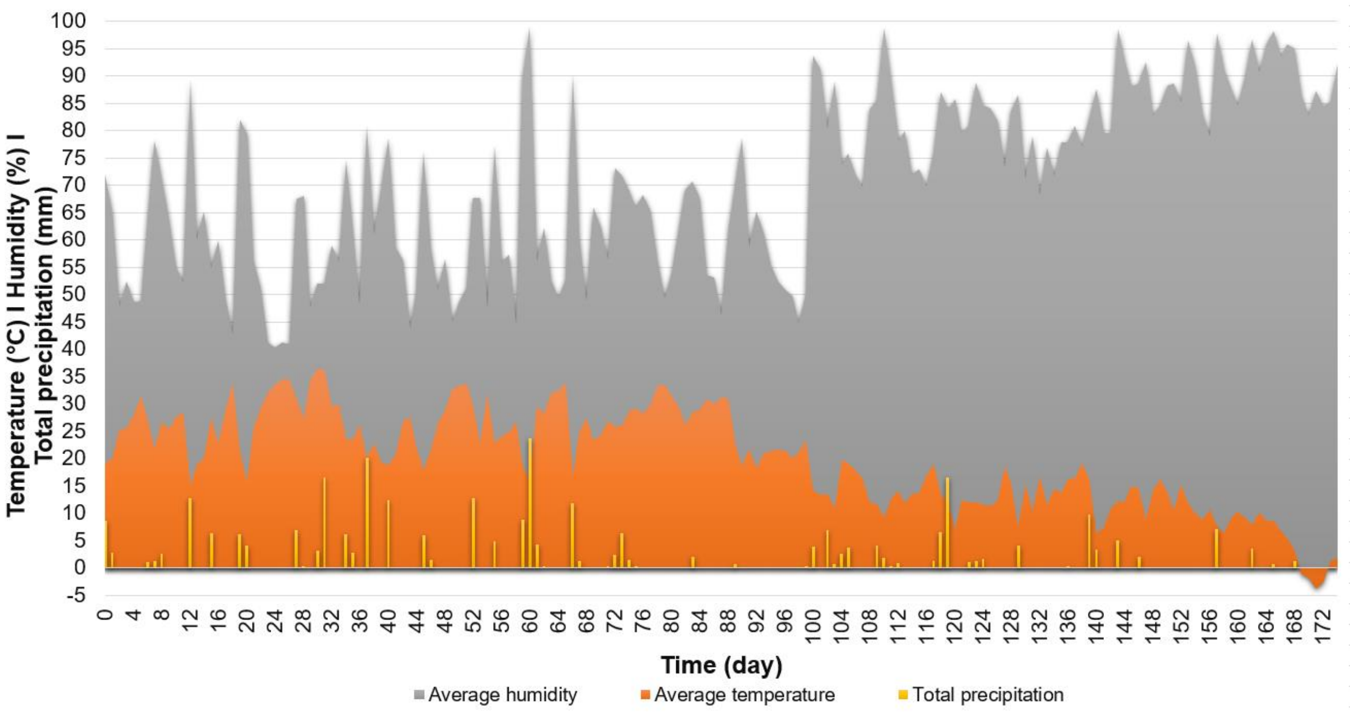

Figure S1. The climate in Warsaw during the tests (1 June 2022 - 30 November 2022).

Table S1. Durability of the hydrophobic and superhydrophobic coatings from the available literature.

Type of coating	Test	Changing in the contact angle	Ref.
■	Exposure to natural conditions, 126 days	From 159.15° to 131.55°	[9]
	Immersion in seawater, 24 h	From 154° to 149° (read from the chart)	[63]
	Immersion in seawater, 24 h	From 154° to 130° (read from the chart)	[63]
	Immersion in solutions with different pH, 30 min	No drop below 150°	[24]
	Immersion in NaCl solution, 120 h	From 168.9° to 153°	[64]
	Immersion in acetic acid solution, 144 h	From 168.9° to 125.3°	[64]
	Immersion in water, 7 days	From 159° to 120°	[65]
	Exposure to natural conditions, 12 weeks	From 159° to 128°	[65]
	Immersion in water, 19 days	From 155.2° to 152.4°	[19]
♦	Immersion in water, 7 days	From 144° to 137°	[26]
	Exposure to natural conditions, 16 month	From 168.2° to 152°	[66]
	Exposure to natural conditions, 35 days	Stable 149° (read from the chart)	[67]

■ Coatings similar to specimens from Hydrophobic Series. Coatings based on anodized aluminum and impregnation.

♦ Coatings similar to specimens from Superhydrophobic Series. Coatings based on a combination of SiO_2 particles and silane or similar ingredients.

Table S2. Icing delay time of coatings from the available literature.

Type of coating	Icing Delay Time	Conditions	Ref.
■	192 s (241 s to snowflake)	-8°C	[68]
	179 s (206 s to snowflake)	-8°C	[68]
	900 s	-20°C	[20]
	250 s	-15°C	[69]
♦	554 s	-5°C	[67]
	>1404 s	-5°C	[67]
	165 s	-20°C	[25]
	276.2 s	-15°C	[57]
	375 s	-20°C	[44]

■ Coatings similar to specimens from Hydrophobic Series. Coatings based on anodized aluminum and impregnation.

♦ Coatings similar to specimens from Superhydrophobic Series. Coatings based on a combination of SiO_2 particles and silane or similar ingredients.

Table S3. Potentiodynamic polarization parameters for hydrophilic, hydrophobic and superhydrophobic coatings from the available literature.

Type of coating	Substrate		Coating		Contact angle	η_P [%]	Ref.
	I_{corr} [A/cm^2]	E_{corr} [V]	I_{corr} [A/cm^2]	E_{corr} [V]			
●	2.72×10^{-5}	-1240	1.51×10^{-10}	-470	n/a	100*	[50]
	6.545×10^{-6}	-631.26	2.373×10^{-6}	-566.74	n/a	63.74*	[70]
	3.136×10^{-6}	-658.67	0.008×10^{-6}	-632.03	n/a	99.74*	[70]
	5.941×10^{-6}	-736.54	0.726×10^{-6}	-726.51	n/a	87.78*	[70]
	4.1×10^{-6}	-581	0.72×10^{-6}	-635	n/a	82.44*	[71]
■	9097×10^{-9}	-1135	2.26×10^{-9}	-328	161.5°	99.98*	[24]
	8.5×10^{-6}	-1682	9.8×10^{-9}	-1360	139°	99.9	[29]
	3.060×10^{-7}	-629	1.527×10^{-9}	-570	155.2°	99.75	[19]
	1.707×10^{-5}	-703	7.734×10^{-8}	-641	158°	99.5	[33]
	curves in article		curves in article		154°	96	[63]
	1.40×10^{-3}	-1130	5.01×10^{-7}	-511	167.7°	99.96*	[35]
	1.65×10^{-4}	-1430	2.36×10^{-8}	-880	170°	99.99*	[72]
	5.65×10^{-6}	-768.6	1.51×10^{-8}	-758.3	163°	99.73*	[73]
	5.65×10^{-6}	-768.6	6.82×10^{-7}	-761.4	hydrophobic	87.93*	[73]
	2.235×10^{-6}	-735	9.333×10^{-9}	-709	162°	99.58*	[20]
	0.95×10^{-6}	-1171	0.11×10^{-6}	-763	93.8°	88.4	[21]
	0.95×10^{-6}	-1171	1.23×10^{-8}	-713	104.9°	98.7	[21]
♦	3.23×10^{-6}	-753	4.64×10^{-8}	-516	167	98.6	[22]
	6.3×10^{-6}	-820	2×10^{-7}	-130	153.2	96.8	[74]
	6.98×10^{-5}	-1450	1.23×10^{-6}	-1110	153	98.24*	[49]
	2.933×10^{-5}	-966	3.744×10^{-10}	-353	155	99.99	[75]
	7.71×10^{-4}	-860	2×10^{-9}	-500	161.35	100*	[76]
	2.5×10^{-4}	-1020	1.4×10^{-11}	-180	174.5	100*	[77]
	6.616×10^{-5}	-711	1.442×10^{-11}	-321	157	100*	[78]

● Coatings similar to specimens from Hydrophilic Series.

■ Coatings similar to specimens from Hydrophobic Series. Coatings based on anodized aluminum and impregnation.

♦ Coatings similar to specimens from Superhydrophobic Series. Coatings based on a combination of SiO_2 particles and silane or similar ingredients.

* calculated based on literature data:

$$\eta_p = \frac{(I_{corr\ subsrate} - I_{corr})}{I_{corr\ substrate}} \cdot 100\%$$

References

9. Olkowicz, K.; Buczko, Z.; Nasiłowska, B.; Kowalczyk, K.; Czwartos J. Superhydrophobic Coating Based on Porous Aluminum Oxide Modified by Polydimethylsiloxane (PDMS). *Materials* **2022**, *15*, 1042.
19. Zheng, S.; Li, C.; Fu, Q.; Hu. W.; Xiang. T.; Wang, Q.; Du, M.; Liu, X.; Chen, Z. Development of stable superhydrophobic coatings on aluminum surface for corrosion-resistant, self-cleaning, and anti-icing applications. *Materials & Design* **2016**, *93*, 261-270.
20. Zang, J.; Yu, S.; Zhu, G.; Zhou, X. Fabrication of superhydrophobic surface on aluminum alloy 6061 by a facile and effective anodic oxidation method. *Surface and Coatings Technology* **2019**, *380*, 125078.
21. Javanpour, B.; Azadbeh, M.; Mozammel, M. Effect of chemical composition of Tetraethoxysilane and Trimethoxy (propyl) Silane hybrid sol on hydrophobicity and corrosion resistance of anodized aluminum. *Silicon* **2020**, *12*, 867-882.
22. Mao, T.; Li, C.; Mao, F.; Xue, Z.; Xu, G.; Amirfazli, A. A durable anti-corrosion superhydrophobic coating based on carbon nanotubes and SiO2 aerogel for superior protection for Q235 steel. *Diamond and Related Materials* **2022**, 129, 109370.
24. Vengatesh, P.; Kulandainathan, M.A. Hierarchically ordered self-lubricating superhydrophobic anodized aluminum surfaces with enhanced corrosion resistance. *ACS applied materials & interfaces* **2015**, *7*, 1516-1526.
25. Zhang, F.; Xu, D.; Zhang, D.; Ma, L.; Wang, J.; Huang, Y.; Chen, M.; Qian, H.; Li, X. A durable and photothermal superhydrophobic coating with entwinned CNTs-SiO2 hybrids for anti-icing applications. *Chemical Engineering Journal* **2021**, *423*, 130238.
26. Amirpoor, S.; Moakhar, R.S.; Dolati, A. A novel superhydrophilic/superoleophobic nanocomposite PDMS-NH2/PFONa-SiO2 coated-mesh for the highly efficient and durable separation of oil and water. *Surface and Coatings Technology* **2020**, *394*, 125859.
29. Jeong, C.; Lee, J.; Sheppard, K.; Choi, C.H. Air-impregnated nanoporous anodic aluminum oxide layers for enhancing the corrosion resistance of aluminum. *Langmuir* **2015**, *31*, 11040-11050.
33. Li, S.; Liu, X.; Li, L.; Zhang, H.; Qiu, C. Drag-reductive and anti-corrosive superhydrophobic surface fabricated on aluminum with thin PDMS/SiO2 coating. *Materials Research Express* **2019**, *6*, 1065a8.
35. Yin, B.; Fang, L.; Tang, A.Q.; Huang, Q.L.; Hu, J.; Mao, J.H.; Bai, G.; Bai, H. Novel strategy in increasing stability and corrosion resistance for super-hydrophobic coating on aluminum alloy surfaces. *Applied Surface Science* **2011**, *258*, 580-585.
44. Zhang, J.; Liu, S.; Huang, Y.; Lv, Y.; Kong, M.; Li, G. Durable fluorinated-SiO2/epoxy superhydrophobic coatings on polycarbonate with strong interfacial adhesion enhanced by solvent-induced crystallization. *Progress in Organic Coatings* **2021**, *150*, 106002.
49. Xie, J.; Hu, J.; Lin, X.; Fang, L.; Wu, F.; Liao, X.; Luo, H.; Shi, L. Robust and anti-corrosive PDMS/SiO2 superhydrophobic coatings fabricated on magnesium alloys with different-sized SiO2 nanoparticles. *Applied Surface Science* **2018**, *457*, 870-880.
50. Telmenbayar, L.; Ramu, A.G.; Yang, D.; Song, M.; Erdenebat, T.O.; Choi, D. Corrosion resistance of the anodization/glycidoxypropyltrimethoxysilane composite coating on 6061 aluminum alloy. *Surface and Coatings Technology* **2020**, 403, 126433.
57. Shen, Y.; Wu, Y.; Tao, J.; Zhu, C.; Chen, H.; Wu, Z.; Xie, Y. Spraying fabrication of durable and transparent coatings for anti-icing application: dynamic water repellency, icing delay, and ice adhesion. *ACS applied materials & interfaces* **2018**, *11*, 3590-3598.
63. Yin ,Y.; Liu, T.; Chen, S.; Liu, T.; Cheng, S. Structure stability and corrosion inhibition of super-hydrophobic film on aluminum in seawater. *Applied Surface Science* **2008**, *255*, 2978-2984.
64. Lomga, J.; Varshney, P.; Nanda, D.; Satapathy, M.; Mohapatra, S.S.; Kumar, A. Fabrication of durable and regenerable superhydrophobic coatings with excellent self-cleaning and anti-fogging properties for aluminium surfaces. *Journal of Alloys and Compounds* **2017**, *702*, 161-170.
65. Saffari, H.; Sohrabi, B.; Noori, M.R.; Bahrami, H.R.T. Optimal condition for fabricating superhydrophobic Aluminum surfaces with controlled anodizing processes. *Applied Surface Science* **2018**, *435*, 1322-1328.
66. Tan, X.; Wang, Y.; Huang, Z.; Sabin, S.; Xiao, T.; Jiang, L.; Chen, X. Facile Fabrication of a Mechanical, Chemical, Thermal, and Long-Term Outdoor Durable Fluorine-Free Superhydrophobic Coating. *Advanced Materials Interfaces* **2021**, *8*, 2002209.
67. Wang, Z.; Gao, X.; Wen, G.; Tian, P.; Zhong, L.; Gou, X.; Guo, Z. Robust silicon dioxide@ epoxy resin micronanosheet superhydrophobic omnipotent protective coating for applications. *Colloids and Surfaces A: Physicochemical and Engineering Aspects* **2018**, *550*, 9-19.
68. Liu, J.; Zhang, X.; Wang, R.; Long, F.; Zhao, P.; Liu, L. A mosquito-eye-like superhydrophobic coating with super robustness against abrasion. *Materials & Design* **2021**, *203*, 109552.
69. Momen, G.; Farzaneh, M.; Jafari, R. Wettability behaviour of RTV silicone rubber coated on nanostructured aluminium surface. *Applied Surface Science* **2011**, *257*, 6489-6493.
70. Abdel-Gawad, S.A.; Osman, W.M.; Fekry, A.M. Characterization and corrosion behavior of anodized aluminum alloys for military industries applications in artificial seawater. *Surfaces and Interfaces* **2019**, *14*, 314-323.
71. Khan, M.F.; Kumar, A.M.; Ul-Hamid, A.; Al-Hems, L.M. Achieving non-adsorptive anodized film on Al-2024 alloy: Surface and electrochemical corrosion investigation. *Surfaces and Interfaces* **2019**, *15*, 78-88.

72. Wang, G.; Liu, S.; Wei, S.; Liu, Y.; Lian, J.; Jiang, Q. Robust superhydrophobic surface on Al substrate with durability, corrosion resistance and ice-phobicity. *Scientific reports* **2016**, *6*, 1-10.
73. Zhang, H.; Yin, L.; Shi, S.; Liu, X.; Wang, Y.; Wang, F. Facile and fast fabrication method for mechanically robust superhydrophobic surface on aluminum foil. *Microelectronic Engineering* **2015**, *141*, 238-242.
74. Shen, Y.; Li, K.; Chen, H.; Wu, Z.; Wang, Z. Superhydrophobic F-SiO2@PDMS composite coatings prepared by a two-step spraying method for the interface erosion mechanism and anti-corrosive applications. *Chemical Engineering Journal* **2021**, *413*, 127455.
75. Tong, W.; Xiong, D.; Zhou, H. TMES-modified SiO2 matrix non-fluorinated superhydrophobic coating for long-term corrosion resistance of aluminium alloy. *Ceramics International* **2020**, *46*, 1211-1215.
76. Gong, A.; Zheng, Y.; Yang, Z.; Guo, X.; Gao, Y.; Li, X. Spray fabrication of superhydrophobic coating on aluminum alloy for corrosion mitigation. *Materials Today Communications* **2021**, *26*, 101828.
77. Zhou, Y.; Ma, Y.; Sun, Y.; Xiong, Z.; Qi, C.; Zhang, Y.; Liu, Y. Robust superhydrophobic surface based on multiple hybrid coatings for application in corrosion protection. *ACS applied materials & interfaces* **2019**, *11*, 6512-6526.
78. Jin, Z.; Mei, H.; Pan, L.; Liu, H.; Cheng, L. Superhydrophobic self-cleaning hierarchical micro-/nanocomposite coating with high corrosion resistance and durability. *ACS Sustainable Chemistry & Engineering* **2021**, *9*, 4111-4121.
